# *In Vitro* Activities of Nisin and Nisin Derivatives Alone and In Combination with Antibiotics against *Staphylococcus* Biofilms

**DOI:** 10.3389/fmicb.2016.00508

**Published:** 2016-04-18

**Authors:** Des Field, Rory O’ Connor, Paul D. Cotter, R. Paul Ross, Colin Hill

**Affiliations:** ^1^School of Microbiology, University College CorkCork, Ireland; ^2^Teagasc Food Research CentreCork, Ireland; ^3^APC Microbiome Institute, University College CorkCork, Ireland; ^4^College of Science, Engineering and Food Science, University College CorkCork, Ireland

**Keywords:** biofilm, bacterial resistance, antimicrobial peptide, nisin, lantibiotic, bacteriocin, staphylococci, antibiotics

## Abstract

The development and spread of pathogenic bacteria that are resistant to the existing catalog of antibiotics is a major public health threat. Biofilms are complex, sessile communities of bacteria embedded in an organic polymer matrix which serve to further enhance antimicrobial resistance. Consequently, novel compounds and innovative methods are urgently required to arrest the proliferation of drug-resistant infections in both nosocomial and community environments. Accordingly, it has been suggested that antimicrobial peptides could be used as novel natural inhibitors that can be used in formulations with synergistically acting antibiotics. Nisin is a member of the lantibiotic family of antimicrobial peptides that exhibit potent antibacterial activity against many Gram-positive bacteria. Recently we have used bioengineering strategies to enhance the activity of nisin against several high profile targets, including multi-drug resistant clinical pathogens such as methicillin-resistant *Staphylococcus aureus*, vancomycin-resistant enterococci, staphylococci, and streptococci associated with bovine mastitis. We have also identified nisin derivatives with an enhanced ability to impair biofilm formation and to reduce the density of established biofilms of methicillin resistant *S. pseudintermedius*. The present study was aimed at evaluating the potential of nisin and nisin derivatives to increase the efficacy of conventional antibiotics and to assess the possibility of killing and/or eradicating biofilm-associated cells of a variety of staphylococcal targets. Growth curve-based comparisons established that combinations of derivatives nisin V + penicillin or nisin I4V + chloramphenicol had an enhanced inhibitory effect against *S. aureus* SA113 and *S. pseudintermedius* DSM21284, respectively, compared to the equivalent nisin A + antibiotic combinations or when each antimicrobial was administered alone. Furthermore, the metabolic activity of established biofilms treated with nisin V + chloramphenicol and nisin I4V + chloramphenicol combinations revealed a significant decrease in *S. aureus* SA113 and *S. pseudintermedius* DSM21284 biofilm viability, respectively, compared to the nisin A + antibiotic combinations as determined by the rapid colorimetric XTT assay. The results indicate that the activities of the nisin derivative and antibiotic combinations represent a significant improvement over that of the wild-type nisin and antibiotic combination and merit further investigation with a view to their use as anti-biofilm agents.

## Introduction

*Staphylococcus aureus* and *S. pseudintermedius* are major human and/or animal pathogens. For humans, *S. aureus* is the leading cause of bacteremia and infective endocarditis as well as osteoarticular, skin and soft tissue, pleuropulmonary, and device-related infections (Tong et al., 2015). It is also one of the main aetiological agents of bovine mastitis, resulting in significant economic losses to dairy enterprises (Viguier et al., 2009). Worryingly, the horizontal transmission of methicillin resistance to *S. aureus* in hospital and community settings, and the growing prevalence of these strains, presents a significant clinical challenge to the management of serious infections worldwide (Stryjewski and Corey, 2014). *S. pseudintermedius* has emerged over the last decade as a critically important, opportunistic animal pathogen responsible for skin, soft tissue, and surgical site infections (Ruscher et al., 2009; Murayama et al., 2013) that also has implications for public health as transmission between humans and animals has been described (Paul et al., 2011). Critically, antibiotic resistance and the ability to form biofilms (complex, sessile communities of bacteria embedded in an organic polymer matrix (Flemming and Wingender, 2010) contribute to the success of *S. aureus and S. pseudintermedius* as pathogens in healthcare, community and veterinary settings. Indeed, biofilm formation is now recognized as an important virulence factor in several *Staphylococcus* sp. (Otto, 2008), providing the bacteria with remarkable resistance to diverse chemical, physical and biological antimicrobial agents and is one of the main causes of persistent infection (Archer et al., 2011). In addition to antibiotic resistance and biofilm formation, the acquisition of other resistance genes and resistance-facilitating mutations in some staphylococci renders these strains impervious to many currently utilized antimicrobial agents (Livermore, 2000; Chambers and Deleo, 2009). As only a handful of new antimicrobials have been developed in the last decade, the further evolution of resistance poses a serious threat to public health. Therefore, there is an urgent need to identify new antimicrobial agents that are not covered by existing mechanisms of resistance. The development of anti-biofilm therapeutics has generally focused on interfering with quorum sensing, inhibition of adhesion, enhancement of dispersion, or bacteriophage-based treatments (Mataraci and Dosler, 2012). Another potential strategy to reduce biofilm-associated resistance is through synergistic effects of antimicrobial agents in combination, which can enhance anti-biofilm activities and help to prevent or delay the emergence of resistance. One such group of compounds with immense potential for therapeutic use is the lantibiotic class of bacteriocins (bacterially derived antimicrobial peptides) (Cotter et al., 2005b, 2013). Lantibiotics are ribosomally synthesized peptides that are defined by the presence of unusual amino acids including lanthionine and/or methyllanthionine (Breukink and de Kruijff, 1999; Chatterjee et al., 2005; Bierbaum and Sahl, 2009). The most meticulously researched lantibiotic is nisin. Produced by *Lactococcus lactis*, nisin exhibits antibacterial activity against a wide range of Gram-positive bacteria, including foodborne pathogens such as staphylococci, bacilli and clostridia. Nisin is used as a food preservative in over 50 countries and has been approved in the EU and by the US Food and Drug Administration (FDA; Delves-Broughton et al., 1996). In addition, both nisin A (and its natural variant nisin Z) are effective against the Gram-positive pathogens responsible for bovine mastitis and have been incorporated into a number of products devoted to restricting or treating such infections (Sears et al., 1992; Wu et al., 2007). Notably, in addition to being effective against planktonic cells of multi-drug resistant staphylococci (Dosler and Gerceker, 2011; Okuda et al., 2013), nisin has also demonstrated efficacy against biofilms (Corbin et al., 2011; Dosler and Mataraci, 2013; Okuda et al., 2013). Moreover, there is also a steadily growing number of engineered nisin peptides that demonstrate enhanced functionalities (activity and/or stability) which make them more attractive from a clinical perspective (Cotter et al., 2013; Field et al., 2015a). Indeed, the bioengineering of nisin has generated variants that exhibit not only improved antimicrobial activity against strains of clinical relevance (methicillin resistant *S. aureus* (MRSA), vancomycin-resistant enterococci (VRE), vancomycin-intermediate *S. aureus* (VISA), methicillin-resistant *S. pseudintermedius* (MRSP), and *C. difficile*) but has also brought about the widening of its antimicrobial spectrum (Field et al., 2008, 2012; Molloy et al., 2013).

It has frequently been suggested that the efficacy of individual lantibiotics could be further boosted through combination with other antimicrobials or membrane-active substances (Cavera et al., 2015a). Indeed, several studies have demonstrated synergistic relationships between conventional antibiotics and lantibiotics (Brumfitt et al., 2002; Cavera et al., 2015b). For example, nisin displayed synergistic activity with the antibiotics colistin and clarithromycin against *Pseudomonas aeruginosa* (Giacometti et al., 2000), with penicillin, streptomycin, chloramphenicol and rifampicin against *Pseudomonas fluorescens* (Naghmouchi et al., 2012) and with ramoplanin and other non-β-lactam antibiotics against many strains of MRSA and VRE (Brumfitt et al., 2002). Similarly, nisin-ceftriaxone and nisin-cefotaxime were found to be highly synergistic against clinical isolates of *Salmonella enterica* serovar Typhimurium as evident by checkerboard and time-kill assays (Rishi et al., 2014). Notably, penicillin, ciprofloxacin and chloramphenicol displayed greater potency against biofilms of *E. faecalis* when used in combination with nisin (Tong et al., 2014). We have previously described a novel nisin variant with improved specific activity compared to nisin A against strains of *S. pseudintermedius* that was also more effective in preventing biofilm formation, and in reducing the biofilm mass formed on microtiter plates (Field et al., 2015b). With this in mind, this study set out to investigate the ability of nisin and two enhanced nisin derivatives (nisin I4V and nisin M21V) in conjunction with a selection of currently utilized antibiotics to control a range of *Staphylococcus* sp., including human and animal associated clinical isolates with the ultimate aim of identifying superior anti-biofilm combinations.

## Materials and Methods

### Bacterial Strains and Growth Conditions

Nisin and nisin derivative producing *L. lactis* strains (**Table 1**) were grown in M17 broth supplemented with 0.5% glucose (GM17) or GM17 agar at 30°C. *E. coli* was grown in Luria-Bertani broth with vigorous shaking or agar at 37°C. A variety of pathogenic staphylococcal targets were selected, including two MRSA clinical isolates (ST528 and ST530), 3 strains associated with animal infections (*S. pseudintermedius* DK729, *S. pseudintermedius* DSM21284, *S. intermedius* DSM20373), 3 isolates associated with bovine mastitis (*S. aureus* DPC5243, *S. aureus* DPC5347, *S. aureus* RF122) and a strain of *S. aureus* (SA113) used as a representative staphylococcal organism in model virulence studies (Kristian et al., 2003; Kneuper et al., 2014; **Table 1**). *S. pseudintermedius* DK729, *S. pseudintermedius* DSM21284 and *S. intermedius* DSM20373 have previously been shown to form biofilms as determined by crystal violet staining (Field et al., 2015b). *S. aureus* SA113 has also demonstrated ability to form strong biofilms (Cramton et al., 1999). *Staphylococcus* strains were grown in cation-adjusted Mueller Hinton (CA-MH) (Oxoid) for minimum inhibitory concentration assays or Tryptic Soy Broth (TSB) (Merck) supplemented with 1% Glucose at 37°C for biofilm assays. Antibiotics were used where indicated at the following concentrations: Chloramphenicol at 10 and 20 μg ml^-1^, respectively, for *L. lactis* and *E. coli.*

**Table 1:** Bacterial strains utilized in this study.

Strain	Relevant characteristics	Reference
*L. lactis* NZ9800 pDF05	*L. lactis* NZ9800 harboring pDF05 (plasmid pCI372 with *nisA* under its own promoter). Wild type nisin A producer	Field et al., 2008
*L. lactis* NZ9800 pDF11	*L. lactis* NZ9800 harboring pDF11 (pCI372-nisA-M21V)	Field et al., 2008
*L. lactis* NZ9800 pDF12	*L. lactis* NZ9800 harboring pDF12 (pCI372-nisA-I4V)	Field et al., 2015b
*S. aureus* SA113	Representative staphylococcal organism in model virulence studies	Kristian et al., 2003
*S. aureus* RF122	Represents the most common *S. aureus* clone derived from bovine mastitis worldwide.	Fitzgerald et al., 1997
*S. aureus* DPC5243	Bovine mastitis-associated strain	Fitzgerald et al., 1997
*S. aureus* DPC5247	Bovine mastitis-associated strain	Fitzgerald et al., 1997
*S. aureus* ST528 (MRSA)	Methicillin resistant *S. aureus* clinical isolate (BSAC)^a^	Piper et al., 2009b
*S. aureus* ST534 (MRSA)	Methicillin resistant *S. aureus* clinical isolate (BSAC)^a^	Piper et al., 2009b
*S. pseudintermedius* DK729	UCC culture collection. Canine pathogen, biofilm former.	Field et al., 2015b
*S. pseudintermedius* DSM21284	Type strain from lung tissue of cat. Biofilm former	Devriese et al., 2005
*S. intermedius* DSM20373	Type strain from pigeon nares. Biofilm former	Hájek, 1976

*^a^British Society for Antimicrobial Chemotherapy.*

### Minimum Inhibitory Concentration Assays

Minimum inhibitory concentration determinations were carried out in triplicate in 96 well microtitre plates as described previously (Field et al., 2010, 2012, 2015b). Briefly, target strains were grown overnight in the appropriate conditions and medium, subcultured into fresh broth and allowed to grow to an OD_600_ of ∼0.5, diluted to a final concentration of 10^5^ cfu ml^-1^ in a volume of 0.2 ml. Chloramphenicol, penicillin G, ampicillin, vancomycin, streptomycin, tetracycline, erythromycin, ceftazidime, and cefuroxime (Sigma) were resuspended in CA-MH media to a stock concentration of 128 or 256 μg/ml. The antibiotics were adjusted to 16, 32, or 64 μg/ml starting concentration and twofold serial dilutions of each compound were made in 96 well plates for a total of 12 dilutions. The target strain was then added and after incubation for 16 h at 37°C and the MIC was read as the lowest peptide concentration causing inhibition of visible growth.

### Nisin and Nisin Variant Purification

Nisin and nisin derivatives were purified according to previously described protocols (Field et al., 2010; Molloy et al., 2013). Briefly, 2 l of Tryptone Yeast (TY) broth were incubated for 20 h with 1% inoculum of an overnight culture of producing strain (nisin A, nisin V, or nisin I4V). This culture was centrifuged for 20 min at 7000 rpm. The supernatant was decanted and passed through 60 g of pre-equilibrated Amberlite XAD16 beads (Sigma–Aldrich). The beads were washed with 500 ml 30% ethanol and eluted with 500 ml 70% isopropanol (IPA) (Fisher) 0.1% trifluoroacetic acid (TFA) (Sigma–Aldrich). Concomitantly, the cell pellets were resuspended in 300 ml of 70% IPA 0.1%TFA and stirred at room temperature for 3 hours followed by centrifugation. This cell supernatant was combined with that referred to above and concentrated through rotary-evaporation (Buchi, Switzerland) to approximately 250 ml. Following pH adjustment to 4.0 further concentration was achieved through the use of a Phenomenex SPE C-18 column to a final volume of 60 ml. 8 ml of this sample was concentrated, through rotary evaporation, to 2 ml and purified through HPLC using a Phenomenex C12 Reverse-Phase (RP) HPLC column (Jupiter 4 μ proteo 90 Å, 250 X 10.0 mm, 4 μm). To facilitate this, a gradient of 30–50% acetonitrile (Fisher) containing 0.1% TFA was developed. The relevant fractions were collected and pooled, subjected to rotary-evaporation to remove acetonitrile and freeze-dried (LABCONCO). The purified peptides were subjected to MALDI-ToF Mass Spectrometric analysis to confirm their purity before use.

### Growth Curve Experiments

For growth experiments, overnight cultures were transferred (10^7^ cfu ml^-1^ in a volume of 1.0 ml.) into CA-MH supplemented with the relevant concentration of nisin wild-type, nisin derivatives, and antibiotic/peptide combinations, and subsequently 0.2 ml was transferred to 96 well microtitre plates (Sarstedt). Cell growth was measured spectrophotometrically over 24-h periods by using a SpectraMax M3 spectrophotometer (Molecular Devices, Sunnyvale, CA, USA).

### Biofilm Formation and Biofilm Treatment With Purified Nisin A, Nisin Derivative, and Antibiotic Combinations

Static microtitre plate assays based on a previous study (Kelly et al., 2012), but with modifications to optimize the assay, were used to investigate the biofilm formation and nisin/antibiotic combination treatments. TSB (Merck) broth supplemented with 1% D-(+)-glucose (Sigma Aldrich) (TSBg) was used in these assays which aids in biofilm formation. Briefly, a 1: 100 dilution was performed by adding 2 μl of log phase cells (10^7^ CFU ml^-1^ of each culture) to 198 μl of TSBg in wells of a sterile 96-well microtitre plate (Sarstedt, Leicester, UK), giving a starting inoculum of 10^5^CFU ml^-1^; 200 μl of TSBg was added to a set of wells as a negative control. All wells were seeded in triplicate. Microtitre plates were then incubated at 37°C for 48 h to allow biofilm formation to occur. After biofilms were established and washed once with phosphate buffered saline (PBS), nisin peptides were added to the microtitre plate wells at 1X, 2X, 4X, 8X, and 16X the relevant MIC as previously determined. All wells were seeded in triplicate. Following incubation for 24 h, at 37°C, the plates were removed and gently washed once with PBS, then 100 μL of a solution containing 500 mg XTT/L (2,3-bis[2-methyloxy-4-nitro-5-sulfophenyl]-2H-tetrazolium-5-carboxanilide) (Sigma) and 10 mM menadione (Sigma) was added to each well. Microtitre plates were incubated for 2 h at 37°C in the dark. Absorbance was measured at 490 nm using a microtiter plate reader (Molecular Devices Spectramax M3, Sunnyvale CA, USA). Data obtained in triplicate were calculated and expressed as the mean ± SD.

### Confocal Microscopy

Biofilms of *S. pseudintermedius* DSM21284 were pre-formed on μ-Plate 96 well uncoated microtitre plates (Ibidi, Germany) suited to confocal microscopy applications. Following peptide treatment, biofilms were rinsed once with PBS and stained by using a Live/Dead BacLight viability kit (Molecular Probes). 100 ml of the solution containing SYTO 9 and propidium iodide mixed in a ratio of 1:1 was added to the biofilm. The films were incubated at room temperature for 15 min in the dark. After incubation, residual stain was removed. The images were observed using a Zeiss LSM 5 exciter confocal microscope with a Plan-Apochromat 63x/1.40 Oil DIC M27 lens and images were acquired using the Zen 2008 SP2 software. Sample were excited using laser light at 488nm with emission light filtered with a bandpass filter at 505–530 nm for Syto 9 and a longpass filter at 650 nm for propidium iodide (PI). Images were acquired using two separate confocal channel (one for Syto 9 and one for PI) with pinhole adjusted to 1 (confocal pinhole) at 1556 × 1556 pixels.

## Results

### MIC-Based Investigations of Antibiotics

MICs for a range of antibiotics, representing penicillins, cephalosporins, glycopeptides, macrolides, aminoglycosides as well as tetracycline, were carried out to establish suitable concentrations for combinatorial studies with nisin and the derivatives nisin V (M21V) and nisin I4V. The MIC was determined to be the lowest concentration of antibiotic that resulted in the absence of visible growth of the target strain after 16 h at 37°C in CA-MH. *S. aureus* SA113, DPC5243, DPC5247, and RF122 proved to be susceptible to the majority of the antibiotics utilized in the study (**Table 2**), with the exception of ceftazidime (CZ). The MRSA isolate ST528 displayed high MICs to erythromycin, chloramphenicol, ampicillin, ceftazidime and cefuroxime (**Table 2**). In contrast, MRSA ST530 remained sensitive to erythromycin and chloramphenicol.

**Table 2:** Minimum inhibitory concentration results of a selection of antibiotics against representative staphylococcal targets.

*Staphylococcus* Strain	Van μg/ml (>2)	Ery μg/ml (>2)	Tet μg/ml (>2)	PenG μg/ml	Cm μg/ml (>8)	Strep μg/ml	Amp μg/ml (>2)	CZ μg/ml (>4)	CF μg/ml (>4)
*S. aureus* SA113	0.488	0.25	0.125	0.0195	4	2.5	0.125	6.20	0.4
*S. aureus* RF122	0.976	0.488	1.0	> 10	2	5	3	16	2
*S. aureus* DPC5243	0.976	0.488	1	0.156	8	5	1	32	4
*S. aureus* DPC5247	0.976	0.488	1	0.156	8	80	2	32	4
**MRSA**
*S. aureus* ST528	0.625	>16	0.25	5	16	40	>64	>32	>32
*S. aureus* ST534	0.625	0.5	0.25	0.312	8	40	8	32	8
***S. pseud/intermedius***									
*S. pseudintermedius*	1.95	>16	4	>10	4	80	32	>32	8
DK729
*S. pseudintermedius*	7.81	>16	>32	0.078	8	>80	0.125	16	0.5
DSM21284
*S. intermedius*	1.95	0.5	4	0.01	2	2.5	0.125	>32	>32
DSM20373

*Staphylococcus pseudintermedius* DK729 and *S. pseudintermedius* DSM21284 both displayed a MIC of >16 μg/ml for erythromycin, while *S. pseudintermedius* DK729 exhibited MICs of 32, >32, and 8 μg/ml for ampicillin, ceftazidime and cefuroxime, respectively (**Table 2**). *S. intermedius* DSM20373 remained sensitive to erythromycin, tetracycline, penicillin and chloramphenicol but displayed high MIC values of >32 μg/ml for both ceftazidime and cefuroxime. These results highlight the multi-drug resistant nature of isolates of *S. intermedius* and *S. pseudintermedius*. Indeed, methicillin resistant *S. pseudintermedius* (MRSP) isolates have been reported that are also typically resistant to aminoglycosides, fluoroquinolones, macrolides, lincosamides, trimethoprim sulfamethoxazol, and, in many cases, to tetracycline and chloramphenicol (Yoo et al., 2010; Yoon et al., 2010).

### Growth Curve-Based Comparisons of the Activity of Nisin A, Nisin Derivatives, and Antibiotic Combinations

**FIGURE 1:**
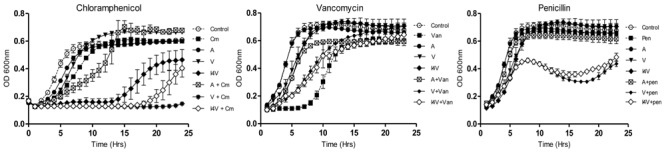
**Growth curve analysis of *Staphylococcus aureus* SA113 in the presence of nisin A **(A)**, nisin V (V), and nisin I4V (I4V) peptides (0.937 μM; 3.0 μg/ml) and in combination with 1.5 μg/ml chloramphenicol (Cm), 5.0 μg/ml vancomycin (Van) and 0.005 μg/ml penicillin (Pen).** The means and standard deviations of three independent determinations are presented.

Having established the MIC values for a range of antibiotics against the representative staphylococci, growth curves were performed in order to reveal more subtle features of the impact of sub-lethal concentrations of nisin A, nisin V, and nisin I4V alone, but also in combination with a selection of antibiotics on bacterial growth. The final concentration of nisin used for each organism was determined on the basis of MIC results obtained previously against these indicator strains (Field et al., 2010, 2015b). One microorganism was chosen from each group to represent drug sensitive strains (SA113), animal associated pathogens (*S. pseudintermedius* DSM21284) and drug-resistant human clinical organisms (MRSA ST528). It was decided that penicillin and chloramphenicol should be included for combinatorial analysis given previous reports of synergism between these antibiotics and nisin A (Lebel et al., 2013; Tong et al., 2014). Indeed, in our studies, a slight increase in lag time was evident when *S. aureus* SA113 was grown in the presence of sub-lethal concentrations of nisin (0.937μM; 3.0 μg/ml) and chloramphenicol (1.5 μg/ml) combined, compared to either compounds used alone (**Figure 1A**). However, a greatly extended lag time was evident in the case of the nisin derivative I4V + chloramphenicol and nisin V + chloramphenicol combinations. In contrast, when nisin and nisin derivatives were combined with vancomycin (0.5μg/ml), no enhanced antimicrobial effect was apparent since the combinations produced a shorter lag time than when vancomycin was used alone (**Figure 1B**). The nisin (0.937 μM; 3 μg/ml) and penicillin (0.005 μg/ml) and nisin derivative/penicillin combinations had little to no effect on the growth of SA113 at the concentrations used (**Figure 1C**).

**FIGURE 2:**
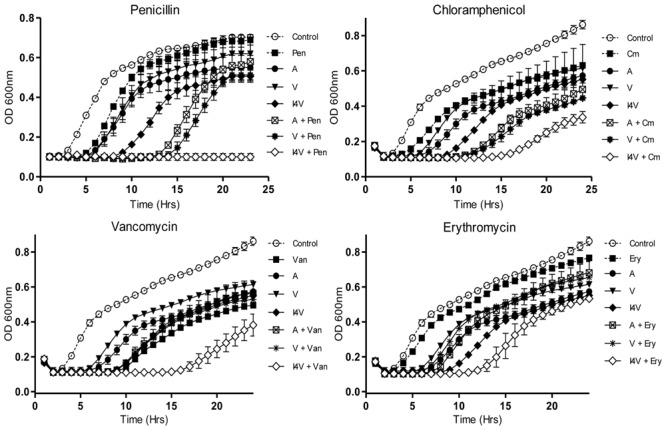
**Growth curve analysis of *S. pseudintermedius* DSM21284 in the presence of nisin A **(A)**, nisin V (V), and nisin I4V (I4V) peptides (0.292 μM; 0.932 μg/ml) and in combination with 0.8 μg/ml penicillin (Pen), 3.0 μg/ml chloramphenicol (Cm), 0.5 μg/ml vancomycin (Van), and 4.0 μg/ml erythromycin (Ery).** The means and standard deviations of three independent determinations are presented.

When *S. pseudintermedius* DSM 21284 was employed, nisin A caused a slight delay in growth relative to the non nisin-containing control at the concentration of peptide used (0.2 μM; 0.6 μg/ml) (**Figure 2A**). Identical concentrations of nisin I4V resulted in a greatly extended lag time, highlighting its greater potency as previously observed (Field et al., 2015b). Furthermore, when combined with penicillin (0.8 μg/ml), the nisin I4V + penicillin combination appeared to completely inhibit the growth of *S. pseudintermedius* DSM 21284. This impact was not apparent for any other combination of nisin or nisin variant (M21V) and penicillin or when any antimicrobial compound was used alone (**Figure 2A**). Indeed, the benefits of employing I4V were evident when combinations of nisin and nisin derivatives with chloramphenicol (**Figure 2B**), vancomycin (**Figure 2C**), and erythromycin (**Figure 2D**) were used in that the longest lag in growth was observed for the nisin I4V and antibiotic combination compared to all others tested.

**FIGURE 3:**
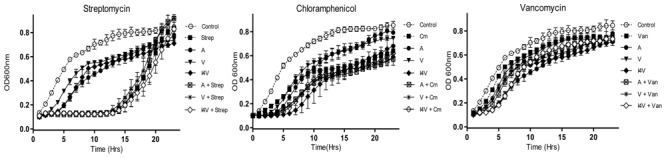
**Growth curve analysis of *S. aureus* ST528 (MRSA) in the presence of nisin A (A), nisin V (V), and nisin I4V (I4V) peptides (0.2 μM) and in combination with 10 μg/ml streptomycin (Str), 2.5 μg/ml chloramphenicol (Cm) and 0.2 μg/ml vancomycin (Van).** The means and standard deviations of three independent determinations are presented.

Infections caused by antibiotic-resistant strains of *S. aureus* have reached epidemic proportions globally. Indeed, the overall burden of staphylococcal disease particularly that caused by methicillin resistant *S. aureus* strains (MRSA), is increasing in many countries in both healthcare and community settings (Chambers and Deleo, 2009). Consequently, we wished to explore the potential of nisin derivative/antibiotic combinations to inhibit the methicillin resistant *S. aureus* ST528 (MRSA) strain chosen as the representative clinical pathogen for combinatorial experiments and growth curve analysis. While the derivatives nisin V and I4V produced a slightly longer delay in growth than the wild type nisin A peptide (0.2 μM), combinations of nisin and the variants with chloramphenicol (2.5 μg/ml) and vancomycin (0.2 μg/ml) did not produce any greater inhibitory effect than when any of the compounds were used alone (**Figures 3B,C**). Similarly, no additional inhibitory effect was observed when penicillin (2.0 μg/ml) was utilized (data not shown) and this trend was also observed when streptomycin (2.0 μg/ml) was applied (**Figure 3A**).

### Investigation of the Anti-Biofilm Activity of Nisin and Antibiotic Combinations

In addition to specific antibiotic resistance, staphylococci have non-specific mechanisms of resistance, of which biofilm formation is undoubtedly the most important (Jain and Agarwal, 2009). Although a number of methods have been developed for cultivation and quantification of biofilms (Stepanović et al., 2007) including the tube test, radiolabeling, microscopy, and Congo red agar plate test, the microtiter plate method remains among the most frequently used assays for investigation of biofilms (O’Toole, 2011). Here, we employed the rapid colorimetric XTT assay to study pre-formed biofilms of *S. aureus* SA113 and *S. pseudintermedius* DSM21284 since this method provides evidence relating to the viability of the remaining biofilm cells following combinatorial peptide/antibiotic treatment which cannot be established via crystal violet staining and because it allows the study of intact biofilms.

**FIGURE 4:**
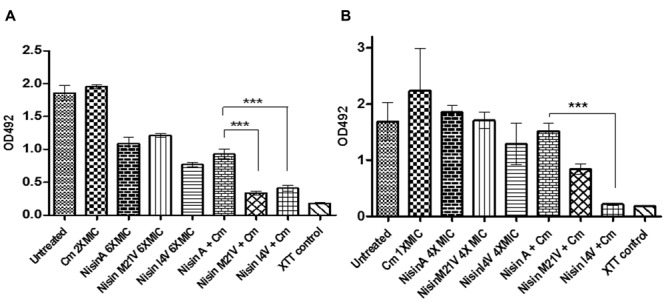
**Viability of biofilms of **(A)***S. aureus* SA113 untreated, and treated with 6X MIC (15μM) of nisin A, nisin V (M21V), and nisin I4V and peptides alone and in combination with 2X MIC chloramphenicol for 24 h and **(B)***S. pseudintermedius* DSM 21284 treated with 4X MIC (1.25 μM) of nisin A, nisin V (M21V), and nisin I4V peptides alone and in combination with 1X MIC chloramphenicol for 24 h as evaluated by XTT (2,3-bis[2-methyloxy-4-nitro-5-sulfophenyl]-2H-tetrazolium-5-carboxanilide) assay measured using a microtiter plate reader.** The means and standard deviations of triplicate determinations are presented. Asterisks indicate statistically significant differences (Student’s *t*-test) between peptide and antibiotic combinations used at similar concentration (^∗∗∗^*p* < 0.001).

In view of the enhanced inhibitory effect of nisin, nisin V, and nisin I4V derivatives + chloramphenicol combinations against vegetative cells of *S. aureus* SA113 (**Figure 1A**), we sought to determine if these combinations could also be effective against pre-formed biofilms. Following preliminary experiments with 1X, 2X, 4X, and 6X MIC of nisin and chloramphenicol alone (data not shown), biofilms of *S. aureus* SA113 formed on a 96-well plate were incubated with nisin and nisin derivative peptides at a concentration of 6X MIC (15 μM; 50.2 μg/ml), chloramphenicol at 2X MIC (8 μg/ml) and combinations thereof for 24 h. The results revealed that while chloramphenicol alone had no significant effect (*p* = 0.2156) compared to the untreated control, nisin, and nisin variants as well as the peptide/antibiotic combinations did result in a significant decrease in biofilm viability compared to the untreated control (**Figure 4A**). Notably, the metabolic activity of biofilms treated with nisin V + chloramphenicol and I4V + chloramphenicol combinations was significantly diminished (*p* = 0.0002 and *p* = 0.0005, respectively) compared to the nisin A + chloramphenicol treatment. Indeed, this result was in agreement with the observed enhanced effect with similar peptide/antibiotic combinations in growth curve analysis against vegetative cells of *S. aureus* SA113 (**Figure 1A**).

**FIGURE 5:**
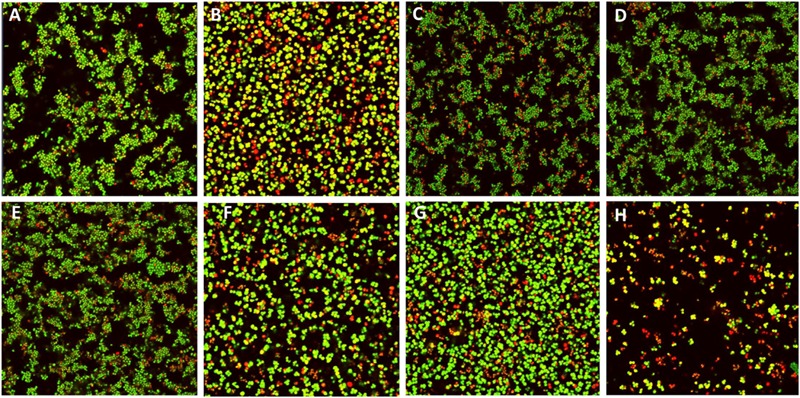
**Live/dead staining confocal images of *S. pseudintermedius* DSM21284 biofilms **(A)** untreated, **(B)** chloramphenicol (Cm) 1X MIC (8 μg/ml), **(C)** nisin A 4X MIC (1.25 μM), **(D)** Nisin V (M21V) 4X MIC (1.25 μM), **(E)** nisin I4V 4X MIC (1.25 μM), **(F)** nisin A + Cm, **(G)** nisin V + Cm, and **(H)** nisin I4V + Cm**.

For nisin treatment alone of pre-formed *S. pseudintermedius* DSM21284 biofilms, a concentration of 4X MIC (1.25μM; 4.19μg/ml) was chosen since previous studies had established that no significant difference was observed in biofilm mass of *S. pseudintermedius* DSM21284 treated with 4X MIC of I4V peptide compared to the wild-type nisin A treated biofilms (Field et al., 2015b). Preliminary treatments of established *S. pseudintermedius* DSM21284 biofilms with 1/2X (4 μg/ml), 1X (8 μg/ml), and 2X MIC (16 μg/ml) of chloramphenicol concentrations revealed that only at 2X MIC was a minor reduction in the biofilm viability observed as determined by XTT assay (data not shown). Thus, for combinatorial experiments, nisin A and nisin derivatives were used alone at 4X MIC, chloramphenicol at 1X MIC and the relevant combinations thereof. Following addition of XTT and optical density readings at 492 nm (OD_492_), no substantial change in biofilm viability was observed following 24 h treatment with nisin A and nisin derivatives alone, chloramphenicol alone, or nisin/chloramphenicol combinations (**Figure 4**) with the exception of nisin I4V + chloramphenicol, where biofilm viability was virtually undetectable compared to the XTT (negative) control (**Figure 4B**). To assess the visual impact of nisin + antibiotic treatments, the experiment was repeated to enable visualization of the treated biofilms using confocal microscopy in conjunction with the BacLight LIVE/DEAD staining kit which facilitates differentiation between active and dead cells (**Figure 5**). The results revealed that the lower fluorescence signalsobserved for the nisin I4V + chloramphenicol treated biofilm compared to that for all other treatments was not due to less metabolic activity attributable to cell death alone, but also as a result of the reduction in biofilm density following treatment (**Figure 5H**).

## Discussion

Staphylococci are commensal bacteria living on the epithelial surfaces of humans and other mammals, and many including *S. aureus, S. epidermidis*, and *S. pseudintermedius*, can cause severe disease when they breach the epithelial barrier. Ominously, antibiotic resistance is widespread in staphylococci, significantly complicating treatment. Moreover, many staphylococci form biofilms, complex structures that confer increased resistance to chemotherapies and host defense mechanisms, making infections difficult to eradicate. Recently, the dramatic rise in antibiotic-resistance has stimulated renewed efforts to identify, develop or redesign existing antimicrobial agents active against these multi-resistant bacteria. Due to their many unique properties, the lantibiotics have become the focus of many biomedical and pharmaceutical research groups due to their demonstrable high potency *in vitro*, multiple modes of action and ability to destroy target cells rapidly (Cotter et al., 2005a; Cavera et al., 2015a). Furthermore, because lantibiotics such as nisin are produced as gene-encoded pre-peptides, they are infinitely more suited than classical antibiotics to bioengineering which could lead to the generation of a new source of potent antimicrobials. Here, we set out to examine for the first time, the ability of nisin and two enhanced bioengineered nisin derivatives (nisin V and nisin I4V) in conjunction with a selection of conventional antibiotics to control a range of *Staphylococcus* sp, including human and veterinary pathogens, with the ultimate aim of identifying superior anti-biofilm combinations. Indeed, following MIC determinations and growth curve analysis in the presence of nisin, nisin V, nisin I4V and selected antibiotic combinations, enhanced inhibitory relationships between nisin + penicillin and nisin + chloramphenicol were observed, but were antibiotic and strain dependent. No synergy was observed for nisin + vancomycin combinations against any of the strains tested. Previous studies have confirmed that synergistic interactions between nisin and vancomycin against MSSA and MRSA can vary widely (Dosler and Gerceker, 2011, 2012). Similarly, nisin + penicillin proved highly efficacious in combination against *S. pseudintermedius* DSM21284 but not *S. aureus* SA113 or MRSA (ST528), while chloramphenicol and nisin combinations proved effective against *S. pseudintermedius* and *S. aureus* SA113 but not *S. aureus* ST528 (MRSA). While this is in agreement with previous reports revealing that nisin and chloramphenicol antagonized each other and did not inhibit the growth of 18 of the 19 MRSA strains tested (Brumfitt et al., 2002), studies involving *E. faecalis* have demonstrated synergism between nisin and chloramphenicol and indeed nisin and penicillin (Tong et al., 2014). Importantly, this synergism also extended to biofilms since confocal laser scanning microscopy revealed that penicillin, ciprofloxacin, and chloramphenicol all displayed stronger anti-biofilm actions in combination with nisin than when these antibiotics were administered alone (Tong et al., 2014). Notably, from the point of view of this study, the derivatives nisin V and I4V both displayed greater potency than nisin A when combined with penicillin and chloramphenicol against *S. aureus* SA113 biofilms. Similarly, nisin I4V + chloramphenicol proved to be the most effective combination against preformed biofilms of *S. pseudintermedius* DSM21284, not only in killing biofilm-associated cells but also in reducing the density of established biofilm (as observed by a decrease in both green and red fluorescence signals). These findings are significant and represent the first report of bioengineered nisin peptides that are improved in combination with specific antibiotics against planktonic or biofilm-associated cells. The findings also further highlight the merits of employing antibiotic combination strategies to enhance the efficacy of available antibiotics, and ultimately, restore sensitivity and reduce their minimum effective dose. These approaches appear particularly promising for combinations of antimicrobials that target different sites. Surprisingly, many studies have shown that the penetration of antibiotics is not lacking in bacterial biofilms. For example, vancomycin diffuses rapidly in biofilms of *S. epidermidis* (Darouiche et al., 1994) and MRSA (Okuda et al., 2013), but exhibits diminished antimicrobial efficacy on bacteria in the biofilm environment. Critically, a number of studies have shown that nisin is bactericidal as a result of its ability to access even the deepest part of a biofilm matrix (Davison et al., 2010; Okuda et al., 2013). Notably, the results generated in this study suggest that specific therapies such as bioengineered nisin peptide and antibiotic combinations may be more efficacious against biofilms, and permit the dose of the individual antimicrobials to be reduced and consequently counter the development of drug-resistance in bacteria. Additionally, the opportunity also exists to combine nisin V and nisin I4V with other antimicrobial agents, including naturally derived compounds including essential oils, diterpenoids and thiazolidinone derivatives that affect biofilms via non-microbiocidal mechanisms, but instead target specific molecular pathways that regulate biofilm formation (Buommino et al., 2014). Furthermore, given that resistance to antimicrobials that target lipid II does not develop easily, combinations of lipid II-targeting compounds may also be indispensable given that it is becoming more and more evident that this essential wall precursor plays a key role in organization of the membrane (Scheffers and Tol, 2015).

From a commercial perspective, it is notable that neither nisin A nor any other lantibiotic is currently employed commercially as a clinical antimicrobial. Its potential with respect to clinical applications is strengthened by laboratory based experiments highlighting its activity against human pathogens, including multi-drug resistant strains (Cotter et al., 2005a; Piper et al., 2009a). Nisin or nisin variants could be applied in the form of a topical therapy as a treatment for generalized bacterial skin infections, and/or used as an adjunct to systemic therapy. Alternatively, nisin could also be an effective inhibitor of biofilms which form on in-dwelling devices or hospital equipment. Notably, although *S. epidermidis* and *S. aureus* have been shown to form *in vivo* biofilms on implanted devices and are the most common pathogens associated with infections of surgical implants and other prosthetic devices, the ability to form a biofilm is only recently gaining attention in the case of *S. pseudintermedius* (Singh et al., 2013).

Although a number of drawbacks pertaining to lantibiotic peptides and their suitability for use as therapeutics are apparent, including low bioavailability and high cost of production, these obstacles may be overcome since a broad range of technologies have been developed for the engineering of lantibiotics. Indeed, the past decade has seen several bioengineering studies describe the generation of peptide derivatives including nisin with enhanced functionality in terms of specific activity, spectrum of activity, solubility and/or temperature and pH stability (Field et al., 2015a). Additionally, genetic systems are in continuous development to increase yields of peptide that may aid commercial viability (Kong and Lu, 2014). The further application of these systems to enhance nisin and other lantibiotics has the potential to lead to the development of novel derivatives for therapeutic use and contribute to a solution to antibiotic resistance across a broad range of bacterial pathogens. In conclusion, we have demonstrated the superior capacity of bioengineered nisin derivatives in combination with classical antibiotics to bring about the destruction of established staphylococcal biofilms of *S. aureus* and *S. pseudintermedius*, which may have future applications for the elimination of problematic biofilms and associated infections.

## Author Contributions

Conceived and designed the experiments: DF, PC, RR, CH. Performed the experiments: DF and RO. Analyzed the data: DF. Contributed reagents/materials/analysis tools: CH and RR. Wrote the paper: DF, PC, and CH.

## Conflict of Interest Statement

The authors declare that the research was conducted in the absence of any commercial or financial relationships that could be construed as a potential conflict of interest.
